# Editorial: Pancreas Imaging Across the Spectrum

**DOI:** 10.3389/fendo.2021.832519

**Published:** 2021-01-03

**Authors:** Amelia K. Linnemann, Vincent Poitout, Guy A. Rutter

**Affiliations:** ^1^ Department of Pediatrics, Herman B Wells Center for Pediatric Research, Indiana University School of Medicine, Indianapolis, IN, United States; ^2^ Indiana Center for Diabetes and Metabolic Diseases, Indiana University School of Medicine, Indianapolis, IN, United States; ^3^ Montreal Diabetes Research Center, Centre hospitalier de l’Université de Montréal, Montreal, QC, Canada; ^4^ Department of Medicine, Université de Montréal, Montreal, QC, Canada; ^5^ Department of Metabolism, Digestion and Reproduction, Imperial College London, London, United Kingdom; ^6^ Lee Kong Chian School of Medicine, Nanyang Technological University, Singapore, Singapore

**Keywords:** islet, pancreas, imaging, *in vivo* imaging, optical imaging, MRI

The pancreas serves two principal roles: the secretion of digestive enzymes from acinar cells and the release of metabolically critical hormones, notably insulin and glucagon, from the endocrine compartment. The latter is formed of discrete structures, the islets of Langerhans, which usually comprise < 2% of the whole mass of the gland. For many years, the bulk of what we knew and understood about the pancreas, and in particular the endocrine pancreas in situ, was garnered primarily from a combination of *ex vivo* analyses of isolated pancreatic islets and post-mortem tissue analyses of rodent pancreata. Recently, however, unprecedented access to human tissues, combined with an ever-evolving myriad of novel techniques for pancreas visualization, have revolutionized our understanding of the pancreas in both normal and diseased states. The *Pancreas Imaging Across the Spectrum* Research Topic contains a collection of review and original research articles that highlight this revolution, and provides a glimpse into the state-of-the-art advances in our understanding of pancreas morphology and function enabled by these technical innovations.

Magnetic resonance imaging (MRI) has long been used for clinical imaging, and its application to imaging pancreatitis has become routine standard of care in the 21^st^ century. In the first article of the Research Topic, Virostko comprehensively discusses recent advances in quantitative MRI and their application to studying the pancreas of individuals with diabetes. Importantly, he highlights efforts to standardize the imaging and processing pipelines to allow efficient quantitative analysis of pancreas heterogeneity, a necessity as the field continues to develop and as large multi-site clinical trials proceed for diabetes treatment and prevention.

Developing a clear understanding of how the pancreas ultrastructure changes during diabetes pathogenesis requires effective analysis of pancreas structure and function in the *in vivo* setting. Many features of the intact pancreas, including both the vasculature and innervation, are disrupted upon isolation of pancreatic islets for analysis. Some of these components remain relatively intact upon isolation of the entire pancreas, but are then disrupted as only a small section of the tissue is processed in most immunohistochemistry or immunocytochemistry analyses. Therefore, much of what we know about pancreas ultrastructure is based on only a partial picture. The fundamental anatomy of the normal pancreas is outlined in a series of papers within this Research Topic. In the first, Gonçalves and Almaça utilize living human pancreas slices from nondiabetic organ donors to demonstrate responsiveness of the human islet vasculature to a variety of stimuli. They couple these observations to parallel analyses of islet calcium oscillations, thus supporting the utility of this approach for valuable insight into pancreas structure-function relationship. Our understanding of human islet microvasculature is further discussed in a review by Dybala and Hara, highlighting both the advances and limitations in the recent literature, and forecasting where the field may be headed using novel technologies. Finally, pancreatic innervation is also disrupted in many approaches, and structure-function analyses have historically been extraordinarily difficult to perform in intact pancreata. Makhmutova and Caicedo discuss this concept in a review article, alongside what we know regarding the complexity of pancreas innervation. They describe novel optogenetic and pharmacological tools that when combined with newly available imaging modalities, have provided significant insight into our basal understanding of pancreas innervation.

Optical clearing methods have been used broadly to study intact tissues and organs, enabling large scale 3D images of tissues in both normal and diseased states. At the level of the whole pancreas, optical clearing approaches are particularly advantageous, and, as described by Campbell-Thompson and Shiue-Cheng Tang, have been bolstered in recent years by the availability of high-powered microscopes capable of imaging the cleared tissue at incredible depth and resolution. These imaging approaches, described as mesoscopic imaging by Alanentalo et al., have contributed significantly to our understanding of pancreas anatomy at both the cellular and molecular level.

Evaluation of pancreatic islet function and plasticity is critical to our understanding of disease pathogenesis. While live cell imaging in the *in vitro* context enables quantitative analysis of dynamic function, this widely used approach is limited by the removal of the islet from its endogenous niche. Disrupting the extensive network of vasculature, innervation, and exocrine spatial relationship with the islets limits the applicability of the observations to relevant physiologic function. Many have turned to islet xenograft and allograft models, including islet transplants into the anterior chamber of the eye, as discussed by Ilegems and Berggren. This site, in particular, has proven to be an excellent location for islet engraftment, enabling revascularization and innervation of the graft, thus allowing longitudinal imaging of islets over long periods of time and providing an unprecedented window into physiologically relevant functional changes during diabetes development.

Finally, the future of clinical application of advances in the field will only be as good as our ability to monitor the effects of novel therapeutics in the *in vivo* setting. Beta cells contain high levels of Zn^2+^, an essential element for the proper processing, storage, and release of insulin. Therefore, Zn^2+^ is of significant interest for the spatial and functional identification of beta cells. Two articles in this Research Topic are focused on the use of agents to monitor zinc release from individual beta cells in the *in vivo* setting. The first, by Chen et al., describes the *in vivo* visualization of a systemically delivered fluorescent granule zinc indicator, ZIGIR. Using this approach, the authors are able to visualize oscillatory release of Zn^2+^ from mouse islet beta cells *in vivo* at cellular resolution. Clavijo Jordan et al. further capitalize upon the use of Zn^2+^ for translational studies in non-human primates, a clear advance towards clinical application of this type of indicator. They utilize a Gd-based zinc sensor, Gd-CP027, as a contrast agent and demonstrate its utility for monitoring functional changes in the pancreas non-invasively using MRI-based imaging.

Collectively, the studies presented within this Research Topic highlight the significant advances afforded by the leaps in imaging capabilities that have been made within the last decade. By applying these technologies, we have now begun to gain an understanding of endogenous pancreas structure and how it relates to function in normal and diseased states. Although important hurdles remain – notably the identification of a robust means to image islet cell mass and hormone secretion non-invasively in man – the furious pace of development in this realm holds significant promise for enabling novel approaches to understand the changes leading up to and after the onset of diabetes and other pancreatic diseases. These seem likely to ensure that the next decade will be just as exciting for moving the field forward.

## Author Contributions

This editorial was written by AL and revised and approved by VP and GR. All authors contributed to the article and approved the submitted version.

## Conflict of Interest

The authors declare that the research was conducted in the absence of any commercial or financial relationships that could be construed as a potential conflict of interest.

## Publisher’s Note

All claims expressed in this article are solely those of the authors and do not necessarily represent those of their affiliated organizations, or those of the publisher, the editors and the reviewers. Any product that may be evaluated in this article, or claim that may be made by its manufacturer, is not guaranteed or endorsed by the publisher.

